# Alcohol Use and COVID-19: Can we Predict the Impact of the Pandemic on Alcohol Use Based on the Previous Crises in the 21st Century? A Brief Review

**DOI:** 10.3389/fpsyt.2020.581113

**Published:** 2020-12-18

**Authors:** Priscila Dib Gonçalves, Helena Ferreira Moura, Ricardo Abrantes do Amaral, João Maurício Castaldelli-Maia, André Malbergier

**Affiliations:** ^1^Department of Psychiatry, Medical School of University of São Paulo (USP), São Paulo, Brazil; ^2^Department of Psychiatry, Universidade Federal do Rio Grande do Sul (UFRGS), Faculty of Medicine, Porto Alegre, Brazil; ^3^Department of Neuroscience, ABC Health University Center, Santo André, Brazil

**Keywords:** alcohol, terrorism, economic crises, COVID-19, pandemic

## Abstract

The enormous health and economic challenges precipitated by the 2019 coronavirus disease (COVID-19) pandemic are comparable or even greater than those associated with previous historical world crises. Alcohol use, especially drinking to cope with stress, is a concern, as an increase in its sales has been reported in some countries during the quarantine. This study aims to provide a better understanding of what to expect in terms of alcohol consumption, risk factors for excessive use, and its potential consequences during this pandemic based on previous experiences. We investigated how traumatic events related to alcohol consumption. Studies on mass traumatic events (i.e., terrorism as 9/11), epidemic outbreaks (i.e., severe acute respiratory syndrome [SARS] in 2003), economic crises (such as 2008's Great Recession), and COVID-19 were selected. The main keywords used to select the studies were alcohol use, drinking patterns, alcohol use disorders, and alcohol-related consequences. Previous studies reported increases in alcohol use associated with those events mediated, at least partially, by anxiety and depressive symptoms, and posttraumatic stress disorder (PTSD). Being male, young, and single also seems to be associated with a higher vulnerability to develop risky drinking behavior after those tragic events. The discussion of previous risk and protective factors can contribute to elaborate more specific public health policies to mitigate the impact of the current pandemic on people's mental health, especially alcohol-related problems.

## Introduction

The 2019 coronavirus disease (COVID-19) pandemic is an unprecedented situation in the 21st century. Since its outbreak, the entire world is facing health and economic challenges. The consequences of this pandemic on people's mental health are still unknown, but the available data suggest that the situation can be considered a “disaster” (1). Disasters like pandemics are collective experiences, also called mass traumas, and quarantine restrictions pose an additional threat to individuals' mental integrity (2).

Anxiety symptoms, mood disturbances, hypochondriac beliefs, poor sleep, and worries are the most common mental health manifestations in the COVID-19 outbreak (3–7). Fear of contamination, personal afflictions (grieving, lack of routine, and isolation), and financial insecurity (i.e., uncertainty and unemployment) are some of the current stressors.

A review of psychological stressful experiences and alcohol intake concluded that stress is associated with increased risk for excessive alcohol use, alcohol-related problems, and alcohol use disorders (AUD) (8). Some recent data showed that alcohol sales and delivery increased during the COVID-19 outbreak (9, 10).

The scientific community has expressed its concern on alcohol misuse during and after the COVID-19 pandemic (10–12) as preliminary studies have been detecting some alcohol-related problems. There are reports of an increased number of emergency room (ER) visits related to alcohol use (including severe alcohol withdrawal syndromes) (13–15) and suicide attempts related to fear of contamination in individuals with severe AUD (16). Additionally, moderate levels of alcohol intake were seen in 28.6% of individuals hospitalized for COVID-19 in England (17).

This narrative review aims to examine the data about the impact of three critical previous disasters on alcohol use. The information extracted from this review will be analyzed as a potential tool to preview the effect of the current crisis on alcohol consumption. We considered the following three previous events: the World Trade Center attack (terrorism), SARS (respiratory epidemic), and 2008's Great Recession. Recent studies on COVID-19 and alcohol use were also reviewed. We run four different searches on PubMed with “alcohol use,” “alcohol-related problems,” or “Alcohol Use Disorder,” and/or “stress,” and/or “PTSD” with the following events separately: 1 World Trade Center or 9/11 attack (terrorism); 2 economic recessions; 3 SARS (respiratory epidemic), and 4 COVID-19 in Title/Abstract. We present the most relevant studies gathered for this review in Table 1, which has been divided accordingly to the following sections: 1 terrorism, 2 economic adversity, 3 SARS, and 4 COVID-19.

**Table 1:** Findings of the main studies included in the present narrative review.

**Author**	**Year**	**Type**	**Section**	**Main findings**	**Limitations**
Beseler et al. (18)	2011	Prospective	1	Drinking motives accessed a decade early predicted greater alcohol use 1 and 16 weeks after 9 /11 in individuals from New Jersey county aged 18–65 years Drinking to cope with negative affect and drinking for enjoyment were the significant variables and no interactions with proximity to the fateful event and history alcohol dependence were noted	Alcohol use was not evaluated right before 9/11
DiMaggio et al. (19)	2009	Meta-analysis	1	An increase in alcohol consumption 2 years after the traumatic event was observed in this meta-analysis that included 31 population-based studies (the majority [24] of studies was from 9/11). These results suggest the need for public health interventions on alcohol use after massive trauma	Heterogeneity of the studies, and a small number of data points inserted in the meta-regression
Hirst et al. (20)	2018	Prospective	1	Findings showed that 1.5% was hospitalized for alcohol- (0.8%) or drug-related diagnosis Participants with PTSD were more likely to have been hospitalized for an alcohol- or drug-related condition than those without PTSD during a period of up to 9 years after 9/11	The study did not include data from federal, psychiatry hospitals out of NY, and emergency department visits
Welch et al. (21)	2014	Longitudinal	1	5–6 years after 9/11, 7.8% of participants reported frequent Binge Drinking (BD) (5+ drinks per occasion, 5+ times in the last 30 days) Higher odds of frequent BD were seen in individuals who were male, young (18–29 years old), never married, smokers, with high school diploma, an income of > 50 K, high exposure of the event and PTSD	The response rate of 68% on wave 2 Self-reported alcohol use and PTSD diagnosis were performed using a self-reported instrument
Welch et al. (22)	2017	Longitudinal	1	10 years after 9/11, 24.6% of the sample reported 1+ episode of Binge Drinking (BD) in the 30 days prior, to those ~37 with high intensity of BD (8+ drinks for men, 7+, women) Higher odds of BD were found in males, younger (18–34 years old), Caucasian, with an income <75 K, higher exposure of the event, and PTSD	Self-reported alcohol use and PTSD diagnosis were performed using a self-reported instrument
Alonso et al. (23)	2017	Longitudinal	2	Data from the National Institute of Statistics (INE, *n* = 21.9 million; 25–64 years) evaluated Deaths Directly Attributable to Alcohol (DDA) and employment status from 2002 to 2011 After the crisis, DDA increased among the employed and decreased among the unemployed, except for men, non-married, and medium/high-wealth people	Only a few DDA were analyzed Alcohol use variables were not available and it some individuals could have history of AUD before the crisis
Ásgeirsdóttir et al. (24)	2014	Longitudinal	2	A random sample of 9,028 individuals from the national survey Health and Well-being, conducted in 2007 and 2009 Reduction (−0.027) in drinking (5+ alcoholic drinks in 1 day at least 1 time/month [past year]) after the crisis among working age population (25–64 y.o.) even after including individual factors as covariates (hours of work, real income, financial assets, mortgage debt, or mental health)	Self-report alcohol use Working age population included a wide age range (25–64 y.o.)
Ásgeirsdóttir et al. (25)	2016	Longitudinal	2	Follow up of the above-mentioned study, conducted in 2012 Reduction of 5% a year in drinking (i.e., 5+ alcoholic drinks in 1 day at least 1 time/month in the past year) during the crisis and at a slower rate (2–3% a year) during recovery among the working-age population, controlling for individual factors (i.e., hours of work, real income, financial assets, mortgage debt, and mental health).	Self-report alcohol use Working age population included a wide age range (25–64 y.o.)
Bor et al. (26)	2013	Longitudinal	2	National survey with >2 million individuals conducted from 2006 to 2010 Frequent binge drinking (4+ episodes in the past 30 days) had a 7% increase, and was associated with young men (< 30 y.o.), not married, non-Black, higher household income, unemployed for <1 year, and without a college degree	Self-report alcohol use
de Goeij (27)	2015	Realistic review	2	Self-medication mechanism could explain a rise in heavy drinking in the US and Spain after the crisis, and that association was stronger in men. Budgetary shortfall could explain the fall in heavy drinking in Iceland	Less evidence for microeconomic (individual) factors
de Goeij (28)	2016	Longitudinal	2	Dutch Health Interview Survey conducted between 2004 and 2013 (*n* = 20, 140 men and 22,394 women aged 25–64) evaluated month-to-month trends in alcohol consumption over several years (episodic [6+ glasses on 1 day 1+ day/week] and chronic drinking [>14 glasses/week for women and >21 for men]) Downward trends showed a ceasing of decline among women in general and among 35–64 and high-income men. A start of decline was observed among younger men (25–34 y.o.)	Self-report alcohol use Harmful drinking was not measured longitudinally (repeated cross-sectional data), and causal relationship cannot be inferred
de Goeij et al. (29)	2017	Longitudinal	2	Data from Dutch Health Interview Survey (*N* = 26,355 aged 30–64 years) collected from 2004 to 2013 Job loss duration (>6 months) was related to both episodic [OR 1.40 (95% CI 1.01–1.94)] and chronic drinking (OR 1.42 [95% CI 1.05–1.91]). Current job loss was associated with chronic drinking (OR 1.43 [95% CI 1.03–1.98]) during the post 2008 economic crisis, but not before. These associations were most clear in men and different between pre-crisis and crisis period (p interaction = 0.023 and 0.035, respectively)	Self-report alcohol use Harmful drinking was not measured longitudinally (repeated cross-sectional data), and causal relationship cannot be inferred
Gili et al. (30)	2013	Longitudinal	2	Primary care patients (*N* = 7,940 in 2006–07 and *N* = 5,876 in 2010–11) were evaluated for mental health disorders AUD diagnosis increased 4.6% (dependence) and 2.4% (abuse) after the crisis. Relative increase, in comparison to other psychiatric disorders, were greater for alcohol dependence and abuse (OR= 12.2 and 4.6, respectively)	Individuals were not evaluated longitudinally
Global Burden of Disease (31)	2016	Longitudinal	2	Between 2000 and 2016 there was a 2% increase in YLD Alcohol was the second behavioral risk factor for YLD and	Inconsistencies in registry data
Kalousova et al. (32)	2014	Longitudinal	2	Data from Michigan Recession and Recovery Study (*N* = 840, followed from 2009–10 to 2011) using Alcohol Use Disorder Identification Test (AUDIT) Harmful drinking was associated with perceived loss of economic resources (HR: 2.75 [95% CI 1.2–6.27] *p* < 0.05), whereas objective measures did not predict this outcome	Data is not nationally representative Objective measures of economic resources were self-reported
Kaplan (33)	2016	Retrospective	2	Data from the U.S. National Violent Death Reporting System (NVDRS) was used to compare heavy drinking among men who committed suicide and living men (Blood alcohol levels [BAC] ≥ 0.08 g/dl for suicide decedents; at least one binge drinking in the last 30 days for the control group) Men who committed suicide had a greater increase (8%) in heavy drinking at the onset of the recession in comparison to living men. For men, adjusted odds ratio was higher after the crisis (adOR =1.15 [95% CI 1.10–1.20; *p* < 0.001]) relative to the prerecession period (adOR = 0.93 [95% CI 0.90–0.97]). The same pattern was not observed in women	BAC measures do not indicate a diagnosis of AUD or harmful drinking Postmortem toxicology testing rates varied across states
Mateo-Urdiales (34)	2020	Longitudinal	2	Data from Spanish Longitudinal Mortality Study (Census) collected from 2004 to 2011 evaluated DDA in a sample of 22.2 million people Largest increase in DDA in men and women with tertiary studies (+ 25.3% and +113.8%, respectively) and smallest in those with primary studies (+6.2% and +1.5%), decreasing relative educational inequalities	Only a few DDA were analyzed Causal relationship cannot be inferred (repetitive cross-sectional data)
Yang (35)	2018	Longitudinal	2	Data from The National Survey on Drug Use and Health (*N* = 307,935) from 2007 to 2016 Millennials were at significantly increased risk of past month binge alcohol (AOR = 1.51; 95% CI = 1.46 ± 1.56) than Gen X, while Baby Boomers were at significantly reduced risk of all substances (AOR = 0.56; 95% CI = 0.54 ± 0.58)	Self-report alcohol use Causal relationship cannot be inferred (repetitive cross-sectional data)
Lancee (36)	2008	Retrospective	3	It was not reported any increase in alcohol intake since the SARS outbreak in Hospital Workers in Canada	Self-report alcohol use
Mak et al. (37)	2009	Retrospective	3	One-third of the sample had psychiatric disorders 30 months after SARS, the most prevalent disorders in this sample were depressive and anxiety disorders, including PTSD. However, the new incidence of AUD was not observed in this infected and hospitalized patients who survived	Small sample size Self-reported questionnaires
Phua et al. (38)	2005	Cross-sectional	3	The use of alcohol and drugs was not observed as a coping mechanism in Healthcare Workers (HCW) in Singapore. Authors stated that cultural e religious factors could contribute to that finding	Small sample size
Wu et al. (39)	2008	Cross-sectional	3	Increased AUD symptoms in hospital employees were related to being male, in quarantining, having a higher household income, working at high-risk locations, high PTS symptoms and depression, hyper-arousal, and drinking to cope, 3 years after the SARS outbreak in China.	It is not possible to determine whether AUD symptoms started before or after the SARS outbreak
Ammar (40)	2020	Cross-sectional	4	An online survey performed in different regions (Europe, Africa, Asia, and Americas) involving 35 institutions showed a decrease in binge drinking during quarantine (*p* < 0.001, d = 0.58) comparing data from 2019 and 2020	Lack of inclusion and exclusion criteria Data from a convenience sample recruited online
Ahmed et al. (41)	2020	Cross-sectional	4	In the overall sample, about one third affirmed the occurrence of anxiety symptoms and 37.1% of depressive symptoms Additionally, 29.1% of the participants reported hazardous drinking, 9.5% harmful drinking, and 1.6% alcohol dependency. Individuals ages 21–40 were more vulnerable to alcohol use Hubei had significantly higher proportions of hazardous drinking (33.5% in Hubei and 21.5% in others); harmful drinking (11.1 vs. 1.9%) and alcohol dependence (6.8% vs. 1.0%).	Self-report scales More than 50% of the sample was from Wuhan province
Fiocruz (42)	2020	Cross-sectional	4	In Brazil, participants were selected using a Respondent-Driven Sampling (RDS) method from April 24th and May 8th. There was a subject perception of increased use of alcoholic beverages by 18% of the respondents, individuals from 30 to 39 years old showed a higher increase. Alcohol intake was associated with feeling sad/depressed (reaching 46.9% of the participants who reported feeling sometimes [22.5%] or always [24.4%] sad/depressed during the pandemic)	Self-report alcohol use Subjective perception of an increase in consumption Data from a convenience sample recruited online
Lee et al. (43)	2020	Cross-sectional	4	Examining the validation of the Obsession COVID-19 Scale (OCS) in the U.S. population, proposing a cutoff point of 7. Findings also showed that higher scores of OCS were correlated with alcohol and drug use to cope	Does not quantify alcohol use
Liang et al. (44)	2020	Cross-sectional	4	Two weeks after the outbreak of the COVID-19 in China, 40.4% of the sample was prone to psychological problems, and 14.4% PTSD symptoms. Among these young adults (age 14–35), those with more negative coping strategies (including alcohol use) had a higher chance of having psychological problems	Self-report Snowball sampling approach
Nanos Research (45)	2020	Cross-sectional	4	Participants were selected using a Random Digit Dialed in April and May. In this Canadian study, among individuals who affirmed staying home more due to COVID-19 (90% of the sample), 20% of those reported increased alcohol, and 21% said they were drinking more often as well. The main reasons for drinking more: lack of a regular schedule, boredom, and stress	Non-standard questionnaire Descriptive analysis
Newby et al. (46)	2020	Cross-sectional	4	Participants filled out an online questionnaire through (March 27th and April 7th), respondents were mostly females (86%). About three-quarters of the subjects said that their mental health was worse. Levels of distress, anxiety, and fears were higher in the respondents with a mental health diagnosis 52.7% declared a hazardous pattern of alcohol use in the prior month (scores ≤ 3/women; ≤ 4 men in AUDIT-C)	Self-report
Stanton et al. (47)	2020	Cross-sectional	4	An online survey carried out between April 9–19, participants were on average 50 years old (SD 14.9) and 67% were women. 22.3% of the respondents affirmed using alcohol 4+ occasions/week, and 26.6% said there was an increase in alcohol use Higher anxiety, depression, and stress levels were noted in individuals aged 18–45 and were related to more elevated alcohol use	Self-report
Sidor and Rzymski (48)	2020	Cross-sectional	4	An online study conducted between 17 April and 1 May (period of national quarantine) in Poland observed an increase of 14.6% in alcohol use. Additionally, individuals who recognized themselves with an AUD reported more frequent alcohol use	Self-report
Sun et al. (49)	2020	Cross-sectional	4	An online survey carried out from March 24–31 observed relapses and an increase in alcohol use during COVID-19 in China. Respondents were on average 28 years old (SD 9) and the distribution of males and females was similar. Results revealed that 32.1% of regular drinkers increased alcohol intake, 18.7% ex-drinkers relapsed, and 1.7% non-drinkers initiated the use of alcohol	Data from a convenience sample recruited online Self-report
Zhang et al. (50)	2020	Cross-sectional	4	The study was conducted between February 28 and March 02 in Wuhan. Participants were submitted to the Perceived Stress Scale (PSS), daily routine, and habits. Results showed that more than 80% reported elevated perceived stress levels. Also, females who were regular alcohol drinkers had more elevated perceived stress levels	Self-report

*1: Terrorist attacks; 2: Economic crises; 3: SARS; 4 Covid-19*.

### Terrorism

In the last 20 years, many studies have been conducted on the effects of terrorism on mental health and alcohol use. A meta-analysis that included investigations on 9/11 twin towers attack, Oklahoma City bombing, and terrorist events in Israel and England showed an increase in alcohol consumption up to 2 years after the traumatic episode. The authors estimated that 7.3% of the population exposed to the event present alcohol misuse after a terrorist attack. The methods used in the researches grouped in this meta-analysis included prospective studies using random digit dial telephone surveys to contact participants, analyses focused on specific groups (i.e., rescue workers, veterans), and longitudinal cohorts, among others (19).

After the publication of this meta-analysis, a community study revealed that drinking motives (i.e., drinking to cope with negative affect and for enjoyment) assessed 10 years earlier could predict greater risk for alcohol use after 9/11 attack (2001) regardless of exposure level to the fateful event and lifetime AUD diagnosis (18).

A unique dataset on the impact of terrorism in society is the World Trade Center (WTC) Health Registry, a cohort of individuals directly exposed to the event. Individuals were assessed at four different time points: Wave 1 (2003–2004), Wave 2 (2006–2007), Wave 3 (2011–2012), and Wave 4 (2015–2016) (51, 52). For this review, two recent studies published by Welch et al. (21, 22) were selected, which investigated the long-term impact of this tragic episode on alcohol use. The authors observed that 7.8% of participants reported frequent binge drinking 5–6 years after 9/11. In addition, frequent binge drinking (5+ drinks per occasion, 5+ times in the last 30 days) was associated with high exposure of the event (4+ experiences such as witnessing terror, being close to someone who died in the event, and others). Higher odds of frequent binge drinking were found in young males (18–29 years old), current and former smokers, with 12–16 years of formal education (high school/college), higher exposure of the event, and participants with posttraumatic stress disorders (PTSD) symptoms. On the other hand, factors related to lower rates of frequent binge were 65 years old or older and being Asian (21).

The second study aimed to investigate the intensity of binge drinking in the previous 30 days, 10 years after the terrorist attack (Wave 3). Their findings revealed that 24.6% of participants reported binge drinking, about one-third of them with high intensity (8+ drinks for men, 7+ drinks, women). Higher odds of excessive alcohol use were observed in young males (18–34 years old), Caucasian, with higher exposure of the event, and with symptoms of PTSD (22).

A more recent study investigated hospitalizations for alcohol- or drug-related diagnosis during a period of up to nine years after 9/11 combining two datasets: the WTC Health Registry and New York State Administrative Hospitalization Data. Six hundred and five individuals (1.5% from a sample of 41,176 subjects) were hospitalized at least once for alcohol- or drug-related diagnosis. Males and individuals with PTSD related to the event were four times more likely to have an alcohol-related hospitalization (20).

Summarizing, risk factors for more frequent and excessive alcohol use were being male and meeting criteria for PTSD. Also, long-term drinking habits related to 9/11 were more likely to occur in younger individuals, with higher exposure (20–22).

### Economic Adversity

Economic crises, *per se*, or as a consequence of other disasters, may affect alcohol use in different ways. At the individual level (micro), alcohol consumption may increase as a way to cope with negative affect. On the other hand, it can decrease due to the loss of economic resources. At the societal/country level (macro), alcohol use may be influenced by public policies (such as social support or preventive strategies for alcohol-related problems), price and availability of alcoholic beverages, and access to treatment (24, 27, 53).

The Great Recession of 2008 was characterized by increased unemployment rates, reduced wages, higher individual debts, and loss of purchasing power. Worldwide countries were impacted and responded differently according to social support (24, 29, 53), alcohol prices (25, 27, 29), and the availability of healthcare services (53, 54), There were some differences also related to regional drinking patterns and cultural and demographic specificities (53).

American studies showed a decline in alcohol consumption during 2008's Great Recession. However, an increase in binge drinking was observed in specific populations, i.e., youngsters, men, unemployed, individuals with fewer years of education, non-Black, and higher income (26, 27, 33, 35, 55). Moreover, subjective perception of economic loss and higher economic adversity in the context of social prejudice were related to problematic drinking in Black Americans and Hispanics (32, 55).

Economic stressors were also relevant to drinking outcomes, and this association was stronger in men (27). A study examined the relation among alcohol use, economic adversity, and suicide. Men who committed suicide had a more significant increase in heavy drinking at the onset of the recession than the male general population. This finding was not observed in female (33).

When comparing alcohol use across generations (Millennials, Generation X, and Baby Boomers), and the impact of the socioeconomic vulnerability, 30) observed that Millennials had an increased risk of binge drinking compared to Generation X, while Baby Boomers had reduced risk. Social vulnerability rates were also higher among Millennials and lower among the oldest cohort, although it was not associated with binge drinking (35).

The Great Recession affected European countries differently. In Spain, higher rates of alcohol-related problems were observed in men, in the working-age population, and those with higher income, whereas results for employment status were mixed. Gili et al. (30) found a significant increase (4.6%) in alcohol abuse and dependence in primary care settings during the recession. A cohort study evaluated Deaths Directly Attributable to Alcohol (DDA) and employment status. Overall, results showed subgroups as unemployed non-married men with substantial material wealth had more unfavorable changes in DDA. At the same time, more favorable outcomes were seen in employed individuals, including unskilled workers (23). A large prospective population study found an increase in DDA after the crisis among men and women in all educational groups. However, this increase was highest in highly educated individuals (+ 25.3% in men and +113.8% in women) and smallest in those with lower education (+6.2% and +1.5%, respectively) (34).

After the Great Recession, economic plans imposed by the European Union, the European Central Bank, and the International Monetary Fund lead Greece to an austerity era. At that time, The Global Burden of Disease Initiative (2018) evaluated years living with a disability (YLD) in the pre- and post-austerity era. From 2000 to 2016, Greece had a 2% increase in YLD, whereas other European countries showed the opposite trend. Alcohol was the second behavioral risk factor for YLD among people aged 15–49 years (31).

In the Netherlands, an epidemiological survey investigated temporal trends for episodic and chronic drinking from 2004 to 2013. Diverted patterns after the crisis suggested that income effect could explain changes in drinking in the lower socioeconomic groups. In contrast, for women and middle-aged high-income men, the self-medication mechanism related to alcohol use was more evident (28). This survey also showed that unemployment was associated with increased alcohol use, especially among men with more extended periods of unemployment (>months). Interestingly, these associations were not found before the crisis (29).

In Iceland, there was a reduction of 5% per year in drinking among the working-age population during the crisis. The devaluation of Icelandic krona (36%) and inflation increased alcohol prices by 48.7% (24). By 2012, Iceland had already recovered from the economic crisis but drinking patterns did not return to its pre-crisis levels and continued to decline at a slower rate (2–3% a year) (25). Therefore, although macroeconomic factors played an important role in reducing drinking, elevated prices could not fully explain this effect. Other variables, such as increased community participation, could have contributed (25).

In conclusion, most studies about economic adversity and alcohol use indicated an increased vulnerability for harmful drinking among unemployed working-age men. Other factors such as marital status, educational background, economic status, psychological distress, ethnic prejudice, and generation also interact with drinking outcomes.

### Severe Acute Respiratory Syndrome (SARS)

The majority of the studies concerning the SARS outbreak assessed healthcare workers' (HCW) risk factors, coping strategies, and the occurrence of mental health problems. One study in emergency department (ED) HCW who assisted patients with SARS in Singapore (38) showed that the main coping strategies were social bonds such as religion and not alcohol/drug use.

Moreover, depressive and anxiety disorders, including PTSD, and not AUD, were the most prevalent disorders in hospitalized patients who survived 30 months after SARS epidemics (37). The authors suggested that the knowledge that alcohol was a risk for Post-SARS avascular necrosis may have been a deterrence to alcohol consumption. Lancee et al. (36) also did not note an elevation in AUD in a Canadian sample affected by SARS.

Contrary to what was observed in the studies above, Wu et al. (39) studied the number of AUD symptoms among hospital employees in Beijing, China, three years after the SARS outbreak. Increased number of AUD symptoms was positively associated with being male, having a higher household income, being quarantined, or working at high-risk locations, as well as drinking to cope, posttraumatic symptoms (PTS), and depression. The relationship between outbreak exposure and AUD symptoms was not affected by sociodemographic factors. Besides, the inclusion of PTS clusters into the model revealed that higher hyper-arousal scores were associated with AUD symptoms.

Three out of four SARS studies examined HCW responses to the epidemic. Increases in alcohol-related problems number of symptoms were significantly associated with higher hyper-arousal scores. Cultural and patient concerns about alcohol-related impacts on SARS could prevent alcohol abuse.

### Coronavirus Disease (COVID-19)

After one semester of the COVID-19 pandemic, several studies reported some increase in alcohol use. In Canada, 20% of the participants who stayed at home increased alcohol consumption. Comparing alcohol use before the pandemic, 21% of the Canadians who stay at home reported drinking more often. The reasons for such behavior were lack of a routine, boredom, and stress (45). A Brazilian study found that 18% of the sample drank more during the pandemic. Participants from ages 30–39 showed the highest increase (25.6%). Alcohol use was associated with feeling sad/depressed (42). In Poland, an increase in alcohol use was seen in 14.6% of the studied sample during quarantine (48). In Belgium, there was a 30.3% increase in alcohol consumption, which was associated with having more children at home, unemployment, and younger age (56). Conviviality was the top motive reported, followed by reward, lack of social contacts, loss of daily structure, and increased tension (56).

In Australia, an online survey administered during the peak of the outbreak (03/27–04/07) revealed that 52.7% of the sample had a hazardous pattern of alcohol use, according to AUDIT-C (46). Another survey conducted between April 9 and 19 showed that ~25% of the adults increased their alcohol consumption mainly due to higher levels of stress, anxiety, and depression symptoms (47). Furthermore, a later online survey, conducted from 4/16 to 5/11, showed that higher levels of stress were associated with harmful alcohol use as well; however, authors reported a decrease in harmful drinking (measured by AUDIT) especially in individuals aged 18–25 (57). Also, Bade et al. (58) observed lower levels of alcohol detection in wastewater analysis in Australia during quarantine in comparison to previous years, suggesting a reduction in drinking among the general population (58). These findings are possibly the result of restrictions to social events associated with drinking behavior (58).

In China, a study in Hubei (the main focus in the beginning of the pandemic) detected higher proportions of harmful/hazardous alcohol use and AUD compared to other provinces (41). In Wuhan (the epicenter of COVID-19), an exploratory study about the living circumstances of those quarantined showed that more than 80% reported elevated perceived stress levels. In this case, women who drink regularly had a two times higher probability of higher perception of stress than abstainers (50) indicating that alcohol and stress could work both ways. Also, in China, individuals reporting more negative coping strategies (including alcohol use) were more likely to have psychological disorders (44). In another study, more persistent thinking of COVID-19 was related to alcohol/drug use as a coping strategy (43). Almost two out of ten ex-drinkers relapsed in youth, and 1.7% started to drink (49).

Despite all studies above reporting an increase of alcohol use during COVID-19, preliminary results of an online international survey (Europe, North Africa, Western Asia, and the Americas) showed that binge drinking decreased in 2020 compared with 2019. One of the possible explanations for this finding was lack of peer pressure in the youth (40).

Most COVID-19 studies show increases in quantity/frequency of alcohol consumption and harmful and hazardous drinking. Boredom, being at home/quarantined, lack of a routine, symptoms of mental disorders, and negative coping styles were associated with those increases. When bored, people want to engage with an activity, but not with whatever is currently available. This conflict is exacerbated when external factors impose restrictions on the range of behaviors they can engage in, which is precisely the current scenario, at a global level, during the period of social isolation in response to the COVID-19 pandemic (59). Struk et al. (60) study suggests that feelings of boredom may contribute to rule-breaking behavior and some negative outcomes, including higher levels of depression and anxiety and problems with alcohol in youth and older adults (61, 62). In that sense, a strong association with drinking and social contact during quarantine was observed in the US (63). This non-adherence to social distancing norms was found among young adults (18–25 y.o) with previous hazardous drinking (63).

The COVID-19 pandemic has also changed alcohol use in adolescents. A Canadian study showed that while there was a decrease in binge drinking, frequency of alcohol use has increased (64). Although alcohol use in adolescence typically occurs in the context of peers, during this pandemic 67% reported solitary drinking. Surprisingly, 93.3% were drinking with their parents, which was also associated with less binge drinking and less use of cannabis or vaping, suggesting a switch to a more “acceptable” behavior when consuming substance at home (64).

However, peer context was still relevant for adolescents and 77.6% reported drinking with friends via technology. More worrisome, 67% reported drinking with friends face-to-face. Concerns of how social distancing would affect their reputation was predictive of face-to-face drinking among those with self-reported low popularity, whereas it was a significant predictor of solitary drinking among those with self-reported high popularity. Depression and fear of infection also predicted solitary drinking (64).

Being home/quarantined requires more organization, self-monitoring, and discipline to accomplish and manage all daily life demands. In this context, some individuals face challenges in setting their routine, having difficulties in discriminating which periods and days are designed exclusively for working, leisure, and household tasks, which can contribute to the increase in their alcohol use as they do not have social restrictions and other immediate negative consequences/reasons related to its use, such as being late to work and underperforming on a meeting among others. Other factors that contribute to alcohol use are social isolation, stress, and negative coping styles, such as drinking to cope with stress and emotion coping. Drinking alcoholic beverages as a mechanism or strategy to tolerate the burden of negative emotions is not recommended and can be unsafe due to its associate with increase in alcohol use and negative alcohol-related outcomes in longitudinal studies (12, 65, 66).

On the other hand, non-adherence to social distancing norms and in-person contacts were also associated with drinking, especially among youth with lifetime hazardous drinking and those with self-perceived low popularity. Therefore, having a structured routine, performing favorable activities, and improving coping skills are considered protective elements to harmful alcohol use and are commonly targeted in alcohol use disorders treatment as well (67). Adolescents may also benefit from interventions aiming to improve self-stem and parents should be advised against the harms of underage drinking.

## Discussion

Individuals respond to traumatic events in different manners, as observed in prior mass trauma situations. Increase in alcohol drinking, especially in specific subgroups, is one of the possible responses (19, 27). Preliminary studies conducted from March to May 2020 indicated an increase in alcohol use, drinking to cope with negative emotions, and depressive and anxiety symptoms (41, 42, 45, 46, 48).

After stressful experiences of terrorist attacks, economic adversity, and epidemics, some sociodemographic characteristics—male gender, unmarried, and young people—seem to predict a higher risk of developing adverse drinking outcomes (higher frequency/quantity, alcohol related-problems). High proximity/exposure to the event is another risk factor (21, 26, 35). These data can help to tailor our preventive strategies to avoid alcohol use problems among the above sociodemographic profile.

On the other hand, in Asia, studies regarding alcohol use and SARS did not show changes in alcohol use patterns. These results could be associated with the use of more adaptive coping strategies (i.e., religion) and less tolerance to alcohol seen in Asian individuals (36, 38, 68, 69).

Various limitations in the current data regarding alcohol use and stressful events should be considered as the lack of standard measures to access alcohol use in those studies. Due to the urgency of the matter, data on alcohol use during the COVID pandemic has been assessed mainly by subjective self-perception of alcohol intake (42, 45, 48). Another frequent limitation in those studies was the lack of quantification of the use of alcohol (43, 44, 70). Concluding, all these variables should be taken into consideration when interpreting the previous study's results and formulating hypotheses for the impact of economic adversities caused by the COVID-19 pandemic.

The full extension of the impact of COVID-19 on mental health is yet to be established. Individuals and regional variables should be considered when developing strategies to mitigate alcohol use problems.

## Author Contributions

PG designed the study, reviewed the literature, edited and critically reviewed the manuscript, and approved the final version of the manuscript. HM reviewed the literature, edited and critically reviewed the manuscript, and approved the final version of the manuscript. RA reviewed the literature, edited and critically reviewed the manuscript, and approved the final version of the manuscript. JC-M edited and critically reviewed the manuscript and approved the final version of the manuscript. AM designed the study, edited and critically reviewed the manuscript, and approved the final version of the manuscript. All authors contributed to the article and approved the submitted version.

## Conflict of Interest

JC-M has been awarded with Independent Grants for Learning and Change (IGLC) from Pfizer (grant IGLC 36893805 and IGLC 37866815), which had no funding relationship with this project. The remaining authors declare that the research was conducted in the absence of any commercial or financial relationships that could be construed as a potential conflict of interest.
